# Effect of Substitutional Metallic Impurities on the Optical Absorption Properties of TiO_2_

**DOI:** 10.3390/nano14141224

**Published:** 2024-07-19

**Authors:** Eduardo Cisternas, Rodrigo Aguilera-del-Toro, Faustino Aguilera-Granja, Eugenio E. Vogel

**Affiliations:** 1 Departamento de Ciencias Físicas, Universidad de La Frontera, Casilla 54-D, Temuco 4811230, Chile; 2 Departamento de Física Teórica, Atómica y Óptica, Universidad de Valladolid, 47011 Valladolid, Spain; rodrigohumberto.aguilera@uva.es; 3 Instituto de Física, Universidad Autónoma de San Luis Potosí, San Luis Potosí 78295, Mexico; faustino@ifisica.uaslp.mx; 4 Donostia International Physics Center (DIPC), 20018 San Sebastian, Spain; 5 Facultad de Ingeniería, Universidad Central de Chile, Santiago 8330601, Chile; eugenio.vogel@ufrontera.cl; 6 Centro Para el Desarrollo de la Nanociencia y la Nanotecnología (CEDENNA), Santiago 7170124, Chile

**Keywords:** DFT calculations, optical absorption properties, electronic properties

## Abstract

(TiO_2_) is both a natural and artificial compound that is transparent under visible and near-infrared light. However, it could be prepared with other metals, substituting for Ti, thus changing its properties. In this article, we present density functional theory calculations for Ti_(1−*x*)_A_*x*_O_2_, where A stands for any of the eight following neutral substitutional impurities, Fe, Ni, Co, Pd, Pt, Cu, Ag and Au, based on the rutile structure of pristine TiO_2_. We use a fully unconstrained version of the density functional method with generalized gradient approximation plus the U exchange and correlation, as implemented in the Quantum Espresso free distribution. Within the limitations of a finite-size cell approximation, we report the band structure, energy gaps and absorption spectrum for all these cases. Rather than stressing precise values, we report on two general features: the location of the impurity levels and the general trends of the optical properties in the eight different systems. Our results show that all these substitutional atoms lead to the presence of electronic levels within the pristine gap, and that all of them produce absorptions in the visible and near-infrared ranges of electromagnetic radiation. Such results make these systems interesting for the fabrication of solar cells. Considering the variety of results, Ni and Ag are apparently the most promising substitutional impurities with which to achieve better performance in capturing the solar radiation on the planet’s surface.

## 1. Introduction

Bulk phases and nanoparticles of titanium oxide (TiO_2_) have received considerable scientific and technological interest, mainly because of the large number of applications in diverse fields. Cosmetics [1], photocatalysis [2], dye-sensitized solar cells [3] and gas sensing [4] are some examples of how these materials have captured researchers’ interest. Even TiO_2_ has been suggested for photovoltaic applications [5,6], despite its wide bandgap allowing electromagnetic absorption just beyond the ultraviolet range, which is practically absent in the solar radiation reaching the surface of the Earth. However, some substitutional impurity types (A) can increase the Ti_(1−*x*)_A_*x*_O_2_ photoreactivity in the visible region, where the solar radiation reaches a maximum power. In fact, even some near-infrared bands could be considered in the development of multi-junction solar cells [7].

The electronic structure of doped TiO_2_ has been studied in previous works [5,8], where the focus has been on the 3d transition metals acting as impurities (V, Cr, Mn, Fe, Co and Ni). For example, a first-principles study considered pristine TiO_2_ in its rutile phase (which exhibits a rigid tetragonal structure with a=4.594 and c=2.959 Å as lattice vectors), while the doped system was in the form Ti_7_A_1_O_16_, which corresponds to a (2 × 2 × 1) rigid supercell [8]. The effects of single 3D transition metal impurity doping the TiO_2_ matrix were studied based on F-LAPW [9] with GGA, as implemented in WIEN97 code [10]. Although the bangap for pristine TiO_2_ was found to be 1.9 eV (much smaller than the experimentally observed value of 3.1 eV [11,12]), the main conclusions were that 3D metal doping creates electronic levels in the pristine bandgap. Moreover, as the atomic number of the dopant increases, these localized levels shift to lower energy values.

Similarly, further first-principles calculations [5] showed that V, Cr, and Fe impurities narrow down the bandgap of pristine TiO_2_ (starting from a calculated value of 2.3 eV for the bandgap), which was corroborated through UV-vis spectrometry, in this way verifying the significant role of ion implantation in the red-shift absorbance spectrum.

Impurities coming from the 3d and 4d series of transition metals were also incorporated in a matrix corresponding to the anatase phase of TiO_2_. The starting point was the calculated pristine bandgap that just reached 2.21 eV. However, the more important qualitative result was that most of the transition metals were able to narrow it down [13], which led to an improvement in the photoreactivity of TiO_2_ in its different forms. When the on-site Coulomb interaction was incorporated through a GGA+U approach in the fist-principles calculations of this system [14], the calculated bandgap agreed with the experimental value (3.21 eV).

The effect of the concentration of Fe impurities in the anatase phase has also been studied, showing that the bandgap of Fe-doped TiO_2_ decreases as the Fe doping level increases. This is an indication of the important changes in TiO_2_’s electronic properties due to the change in the orbitals following substitutional impurity.

Bandgaps for the rutile phase that are closer to the experimental values can be calculated by including on-site Coulomb corrections. For example, by including corrections between 3d orbital electrons of Ti atoms and the 2p O orbital electrons under the LDA+U formalism [15], it was possible to obtain a bandgap of 3.1 eV, which is quite close to the experimental value [11,12]. In this manner, it was also shown that correction parameters affect only the behavior of the conduction band, while values higher than 7 eV only allow for an unphysical description of the electronic interactions. The results are dramatically improved when correlation corrections are additionally introduced to the O 2p orbitals.

In this work, we will investigate the electronic, structural, and geometrical properties of TiO_2_ bulk systems with neutral substitutional impurities. We will focus on the following impurity types: 3d transition metals (Fe, Co Ni) and noble metals (Cu, Ag, Au, Pd, Pt). Such a selection allows for a systematic study on the effect of element groups 10 and 11, while Fe and Co act as references for our calculations. In Section 2, we present the theoretical approach and computational details corresponding to the structural relaxation of the system, which includes on-site Coulomb corrections under the LDA+U formalism. Section 3 is devoted to the results (structural, density of states, and optical results) and their corresponding discussion. Finally, in Section 4 we summarize some conclusions.

## 2. Theoretical Approach and Computational Details

Our approach to this investigation was a two-step process beginning from the density functional theory (DFT) level. We first used the Spanish Initiative for Electronic Simulations with Thousands of Atoms (SIESTA) [16] code to perform pre-optimization of the structure, obtaining reliable lattice constants, regardless of the electronic states at this point. Then, we invoked the QUANTUM ESPRESSO v6.3 [17] package to perform definite structural relaxation (starting from the coordinates obtained with SIESTA), now including Hubbard’s corrections to account for the bandgap in our pristine metallic matrix oxide.

For the pre-optimization, we used the free distribution computational package SIESTA v4.0 to carry out density Ffunctional theory (DFT) calculations following the generalized gradient approximation (GGA) proposed by Perdew–Burke–Ernzerhof (PBE) [18]. SIESTA makes use of numerical pseudo-atomic orbitals as basis sets to solve single-particle Kohn–Sham equations. In this way, the atomic cores are approximately described by nonlocal norm-conserving Troullier–Martins pseudopotentials [19] factorized in the Kleinman–Bylander form [20]. The pseudo-potentials for O, Ti, Fe, Co, Ni, Cu, Pd, Ag, Pt and Au were obtained by considering the following valence configurations: $2s^{2}2p^{4}$, $4s^{2}3p^{6}3d^{2}$, $4s^{2}3d^{6}$, $4s^{2}3d^{7}$, $4s^{2}3d^{8}$, $4s^{1}3d^{10}$, $5s^{1}3d^{9}$, $5s^{1}4d^{10}$, $6s^{1}5d^{9}$, and $6s^{1}5d^{10}$, respectively. Double-ζ doubly polarized basis sets were employed to describe the valence states. Further details on the pseudopotentials and basis sets used for the impurities, as well as on the pertinent tests, can be found in our previous works [21,22,23].

**Figure 1:**
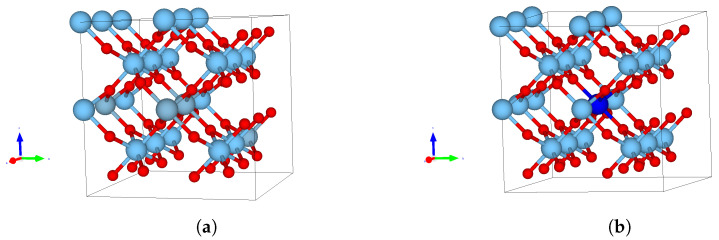
(**a**) 2 × 2 × 3 unit cell modeling pristine TiO_2_ in the rutile phase (Ti_*m*_O_*n*_) with m=24 and n=48. Ti (O) atoms appear represented in light blue (red). (**b**) A substitutional metallic impurity (blue ball) leading to Ti_*m*−*l*_A_*l*_O_*n*_. In particular, l=1 corresponds to an impurity concentration of 4.16%.

We performed structural relaxation under both a spin-polarized framework and a paramagnetic regime to address possible magnetic behavior. Additionally, we considered a 2×2×3 unit cell of TiO_2_ (see Figure 1a), which is equivalent to a Ti_*m*_O_*n*_ super-cell (m=24, n=48). By imposing periodic boundary conditions, we mimicked pristine bulk TiO_2_, which can be modified by metallic substitutional impurities, leading to Ti_(*m*−*l*)_A_*l*_O_*n*_, where A can be Fe, Co, Ni, Cu, Pd, Ag, Pt, or Au (see Figure 1b). In what follows, we focus on l=1, which corresponds to an impurity concentration of 1/24=4.16%.

## 3. Results and Discussion

### 3.1. Structures and Energies

Table 1 summarizes the results for the relaxed structure, including the lattice constants a, b, c, c/a ratio, magnetic moment per unit cell, and energy difference per atom, depending on whether or not magnetic corrections were included. As can be observed, only slight variations were found in the lattice constants due to the presence of one impurity atom of any kind. This is not surprising since Table 1 contains values for the whole cell.

A host of differences were found for the magnetic moments per unit cell, ranging from 0 in several of them to 2.5 for iron.

Particularly notorious is the case of copper with 2.0 $\mu_{B}$/cell. In general, magnetism does not play an important role in these systems, in which the magnetic moment for 4D and 5D metals is zero, just as in the case of the nickel system. Only in the case of iron do cobalt and copper show magnetism.

The magnetic moment per cell reported in Table 1 is of relative importance for Fe, Co and Cu only. This is a magnetic moment shared by the 72 atoms of the cell, so it has little weight in terms of the magnetic properties of the system.

Further calculations show that a paramagnetic configuration minimizes the total energy for the Ti_23_CuO_48_ and Ti_23_AuO_48_ systems. In fact, by defining the total energy difference per atom as $\Delta \epsilon_{p}=\left(E_{p}-E_{s}\right)/\left(l+m+n\right)$, where $E_{p}$ and $E_{s}$ correspond to the total energies in a paramagnetic and spin-polarized calculation, respectively, we can observe that thermal effects at room temperature (25 meV) are enough to demagnetize any of these systems (see the last column of Table 1). Even when the ferromagnetic behavior is observed experimentally for Cu-doped TiO_2_, the negligible remanent magnetization is in agreement with our results [24,25]. For these reasons, we concentrate on the calculation of properties based on the energy bands in the paramagnetic regime.

We also determined the formation energy for Ti_(*m*−*l*)_A_*l*_O_*n*_, which is defined as follows [21,23,26,27,28,29,30]:

(1) $$E_{\mathrm{Formation}}\left[{\mathrm{Ti}}_{m-l}A_{l}O_{n}\right]=E_{\mathrm{Total}}\left[{\mathrm{Ti}}_{m-l}A_{l}O_{n}\right]-E_{\mathrm{Vac}}^{*}\left[{\mathrm{Ti}}_{m-l}O_{n}\right]-lE_{\mathrm{Atom}}\left[A\right].$$

In this equation, A stands for the element acting as a substitutional impurity in pristine TiO_2_, $E_{\mathrm{Total}}\left[{\mathrm{Ti}}_{m-l}A_{l}O_{n}\right]$ corresponds to the total energy of the system, $E_{\mathrm{Vac}}^{*}\left[\;{\mathrm{Ti}}_{m-l}O_{n}\right]$ representing the total energy of the system presenting *l* vacancies of Ti, and $E_{\mathrm{Atom}}\left[A\right]$ is the energy of an isolated atom of element A. In what follows, we have m=24 and n=48, and l=1. Thus, for A = Ti, we can obtain the formation energy of pristine TiO_2_.

In a similar way, we found the binding energy, which is defined as follows:

(2) $$E_{\mathrm{Bind}}\left[{\mathrm{Ti}}_{m-l}A_{l}O_{n}\right]=E_{\mathrm{Total}}\left[{\mathrm{Ti}}_{m-l}A_{l}O_{n}\right]-lE_{\mathrm{Atom}}\left[A\right]-\left(m-l\right)E_{\mathrm{Atom}}\left[\mathrm{Ti}\right]-nE_{\mathrm{Atom}}\left[O\right].$$

Using Equation (2), we obtained the binding energy of pristine TiO_2_ when l=0 (m=24 and n=48), and thus we computed the delta binding energy per atom as follows:

(3) $$\Delta E_{\mathrm{Bind}}=\frac{E_{\mathrm{Bind}}\left[{\mathrm{Ti}}_{m-l}A_{l}O_{n}\right]-E_{\mathrm{Bind}}\left[{\mathrm{Ti}}_{m}O_{n}\right]}{l+m+n}$$

**Figure 2:**
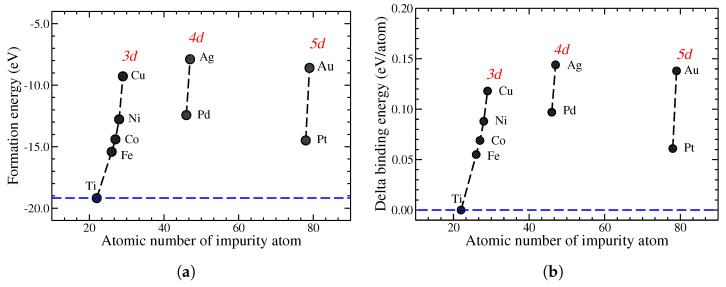
(**a**) Formation energy and (**b**) delta binding energy as a function of the atomic number of the metallic impurity in TiO_2_. The horizontal dashed line corresponds to pristine TiO_2_.

The formation energies and delta binding energies per atom for all the systems under study are shown in Figure 2a and Figure 2b, respectively. These are presented as a function of the atomic number of the metallic impurity, while in the pristine case, they depicted by a horizontal dashed line. In both cases, we observe agreement with previously computed values for a (TiO_2_)_10_ cluster presenting substitutional impurities [23].

### 3.2. Electronic Density of States

Considering the low demagnetization energy barriers reported in Table 1, in what follows, we concentrate on the paramagnetic regime of each system. Of prime importance is the calculation of the Fermi level ($E_{Fermi}$), which will be used as the reference energy in the forthcoming diagrams. The calculated DOS curve is integrated up to the point when the number of electrons obtained coincides with the total number of electrons in the cell. The maximum energy reached in the integration process corresponds to $E_{Fermi}$.

**Figure 3:**
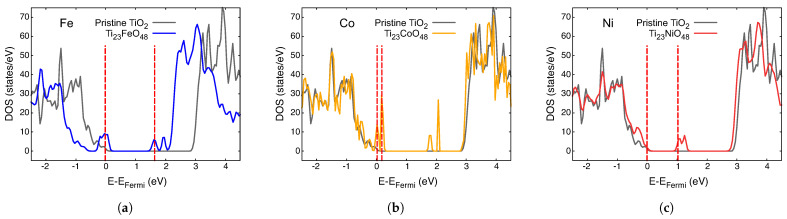
Density of states of paramagnetic Ti_23_AO_48_: (**a**) A = Fe; (**b**) A = Co and (**c**) A = Ni. The DOS for pristine TiO_2_ is plotted gray in each case. The vertical red dashed lines are visual guides with which to identify the bandgap.

**Figure 4:**
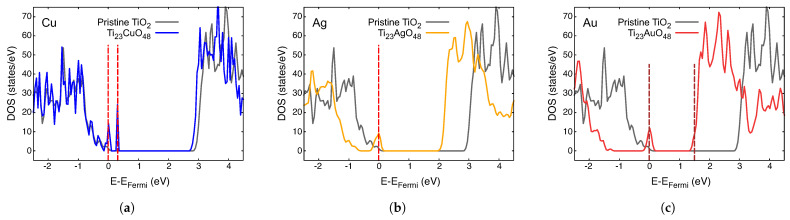
Density of states of paramagnetic Ti_23_AO_48_: (**a**) A = Cu; (**b**) A = Ag; and (**c**) A = Au. The DOS for pristine TiO_2_ is plotted in gray in each case. Vertical red dashed lines are visual guides with which to identify the bandgap.

**Figure 5:**
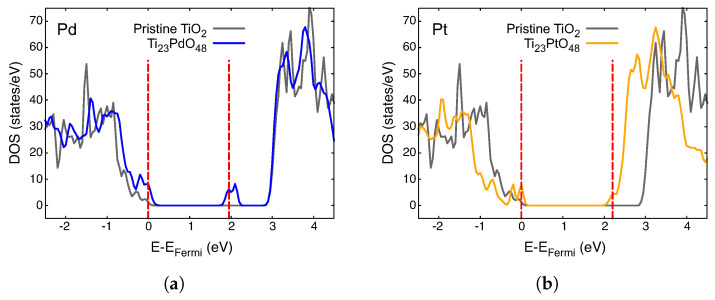
Density of states of paramagnetic Ti_23_AO_48_: (**a**) A = Pd; (**b**) A = Pt. The DOS for pristine TiO_2_ is plotted in gray in each case. The vertical red dashed lines are visual guides with which to identify the band gap.

The density of states (DOS) for different impurities, A, in Ti_23_AO_48_ is presented in different colors in Figure 3, Figure 4 and Figure 5. The total DOS for pristine TiO_2_ is depicted in the gray curve. The vertical red dashed lines indicate the Fermi level (left) and the first unoccupied state (right) in each case, and they are mere visual guide with which to identify the bandgap. We now discuss these figures collectively.

The first general observation is that new, mostly narrow levels appear in the gap in all these cases, which indicates an effective decrease in the bandgap compared with that of pristine crystal. The case of Ni is probably the simplest to analyze; consider Figure 3c, showing the Fermi level just at the top of the lower band. Impurity-empty levels appear around 1 eV above $E_{Fermi}$, which is a clear indication of the lower energy absorption possibilities, as we will discuss in the next section.

For all the other metals in the preceding figures, $E_{Fermi}$ lies on an impurity band or just under it. The presence of empty levels just over $E_{Fermi}$ creates new dynamics for these systems, which is important for their optical properties.

### 3.3. Optical Absorption Properties

The electromagnetic absorption spectrum is obtained by applying linear optical response approximation. In this way, the imaginary part of the dielectric function, $\varepsilon_{i}\left(\omega \right)$, is related to the optical absorption coefficient, κ(ω), through the following equation:

(4) $$\kappa \left(\omega \right)=\frac{\omega \varepsilon_{i}\left(\omega \right)}{c\;n(\omega )}\;,$$

where ℏω corresponds to the incident photon energy, *n* is the refractive index of the pristine material, and *c* is the speed of light. The *epsilon.x* toolset, a post-processing code available in the Quantum Espresso [17] v6.3 package, is employed to obtain the complex dielectric function. Thus, in the framework of band theory without electron hole interaction, $\varepsilon_{i}\left(\omega \right)$ can be obtain using Drude–Lorentz equations [31,32,33]. Such approximation is enough to obtain general trends of the optical properties of the doped system, so we estimate more sophisticated treatments using the GW Bethe–Salpeter equation [34].

**Figure 6:**
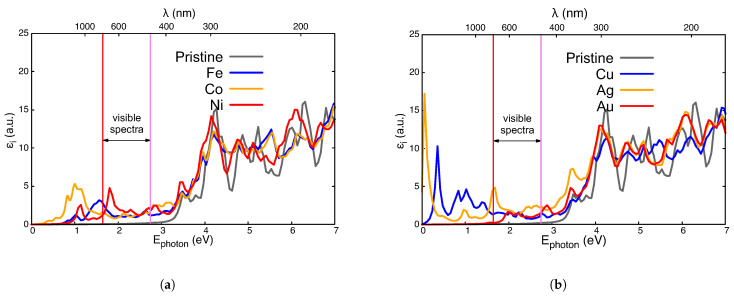
Imaginary part of the dielectric functions, $\varepsilon_{i}\left(\omega \right)$, for Ti_23_AO_48_ compared with those for pristine TiO_2_ (gray line). (**a**) A = Fe (blue line); A = Co (yellow line); and A = Ni (red line). (**b**) A = Cu (blue line); A = Ag (yellow line); A = Au (red line). Red and violet vertical lines are included to highlight the visible spectra.

Figure 6 shows the imaginary part of the dielectric function, $\varepsilon_{i}$, obtained for the paramagnetic regime of Ti_23_AO_48_: A = Fe, Co, Ni (Figure 6a); A = Cu, Ag, Au (Figure 6b). Any one of these substitutional impurities improves the electromagnetic absorption of pristine TiO_2_ (whose $\varepsilon_{i}$ is depicted in gray). This is especially relevant for the visible spectrum (between two vertical lines) and the adjacent near-infrared transition because of their possible applications in photovoltaic technologies.

The Fermi level of the Ti_23_FeO_48_ system lies at the center of a narrow impurity band, thus allowing absorptions from the valence band (VB) approximately below 1.5 eV (Figure 3a). Absorptions of a higher energy (with that of a broad structure at around 1.6 eV in Figure 6) receives contributions originating from lower valence states and also from those ending in impurity levels under 2 eV at the top of Figure 3.

The imaginary part of the dielectric function for Ti_23_CoO_48_ (also in Figure 6a) shows that the first band more is abundant and displaced to lower energies, which is due to the several impurity levels just over $E_{Fermi}$ and the proximity of states in VB.

The spectrum of Ti_23_NiO_48_ in Figure 6a presents two leading structures at about 1.1 eV and 1.8 eV. The former is sustained via transitions from the edge of the VB to the impurity doublet level at 1 eV. Next, we find a broader and more pronounced structure centered at 1.8 eV corresponding to transitions from more abundant, deeper states at the VB. In fact, these last transitions are very desirable for solar cell applications.

The imaginary parts of the dielectric functions for the Ti_23_CuO_48_, Ti_23_AgO_48_ and Ti_23_AuO_48_ systems are presented in Figure 6b. Cu presents near-infrared absorptions because of impurity levels both below and above $E_{Fermi}$. The case of Ti_23_AuO_48_ presents a similar level of activity for the visible range due to transitions from the edge of the VB to this impurity level, which leads to a weak imaginary part of the dielectric function at low or intermediate energies (Figure 6b). The case of Ti_23_AgO_48_ is like that with a Au impurity, with a very important difference: the edge of the VB is nearer the impurity level in the pristine gap, which leads to important optical activity in the red and infrared zone of the spectrum.

**Figure 7:**
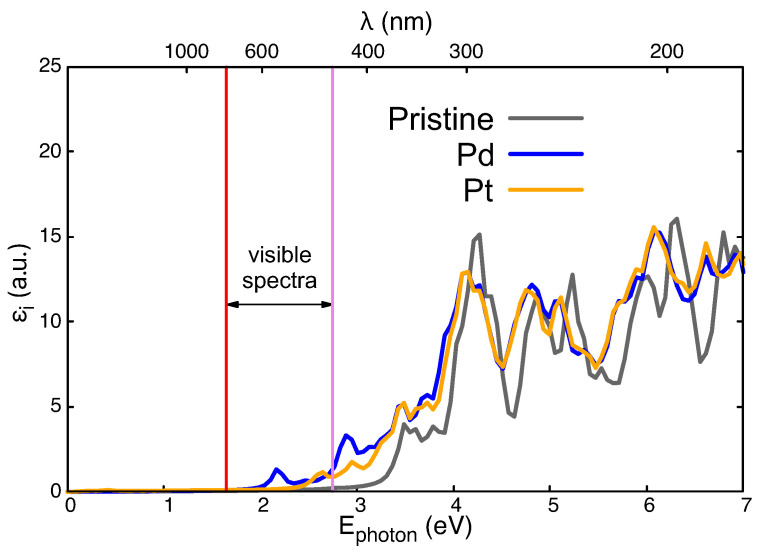
Imaginary part of the dielectric functions, $\varepsilon_{i}\left(\omega \right)$, for Ti_23_AO_48_ compared with pristine TiO_2_ (gray line). The cases of A = Pd and A = Pt are depicted by the blue and yellow lines, respectively. Red and violet vertical lines are included to highlight the visible spectra.

Optical results for Pd and Pt are presented in Figure 7, which was created using the same techniques as those used for the previous figure. It can be observed that changes in the pristine case are not as important as those in previous cases, probably due to the lower hybridization of these 4D and 5D electrons in these wider atoms. Therefore, no activity in the infrared zone is to be found in any of these two systems. The presence of an impurity doublet just under 2 eV in Figure 5a explains the structure within the visible spectrum in Figure 7 for Pd (4D atomic configuration). No such impurity level is seen for Pt in Figure 5b, meaning that there is a weaker and blue-shifted absorption spectrum for Pt (5D atomic configuration in Figure 7).

Since the impurities could have had different charge states [35], we obtained the absorption spectrum for charged impurities in the case of Ni and Ag, both of which exhibit absorption peaks in the visible spectrum. As shown in Appendix A, positively charged impurities shift the absorption peaks to higher energies, although they remain within the visible spectrum. However, particularly for Ni, negatively charged impurities degrade the optical absorption properties. Thus, it is necessary to extend the present study to include the different charge states of the impurities [36].

## 4. Conclusions

The first general conclusion is that the substitution of Ti by neutral metal elements in TiO_2_ introduces electronic levels within the pristine energy gap. The density of states is different for each impurity, which provides several different channels for the possible applications.

The new energy gap can be very low (Cu, Ag, Co) or relatively wide (Pd, Pt). Intermediate energy gaps are obtained for Fe, Ni and Au. Such results are a consequence of the change in the overlap between the *d* states of the impurity with the 2p states of oxygen. Then, the introduction of energy levels within the pristine compound gap is possible, bringing in new electronic states that can enhance photocatalytic activity.

Quite relevant is the absorption activity in the visible and near-infrared spectrum, which can improve the performance of these materials such that they become good solar energy absorbents at room temperature. Therefore, Ni, Ag, Fe, Cu, and, to a lower extent, Au are good candidates for the visible or near-infrared absorption spectrum, as shown in Figure 6.

The optical absorption peaks of different impurities indicate the potential of their applications in multi-junction solar cells, which involve the stacking of materials with different bandgaps to absorb different frequencies of the visible spectrum, showing intense photo-response behavior.

In any case, our results reflect tendencies rather than exact values, so larger simulations with different concentrations (and large computer facilities) are needed to obtain more precise results. On the other hand, the different charge states of impurities must also be taken into account, which can be analyzed in future theoretical work. Having declared the limitations, we suggest Ni as the best candidate with which to substitute Ti in TiO_2_ to test this proposal in real experiments.

## Figures and Tables

**Table 1:** Lattice constants, magnetic moment and $\Delta \epsilon_{p}$ for different substitutional metallic impurities on TiO_2_.

Impurity	a	b	c	c/a	Magnetic Moment	$\Delta \epsilon_{p}$ ^(a)^
	Å	Å	Å		$\mu_{B}$/Cell	meV
Pristine	4.621	4.621	3.055	0.661	0.0	-
Fe	4.609	4.609	3.046	0.661	2.5	6.1
Co	4.602	4.647	3.046	0.662	1.0	1.0
Ni	4.603	4.602	3.046	0.662	0.0	-
Cu	4.605	4.605	3.058	0.664	2.0	−9.4
Pd	4.617	4.617	3.058	0.662	0.0	-
Ag	4.626	4.756	3.063	0.662	0.0	-
Pt	4.617	4.617	3.060	0.663	0.0	-
Au	4.628	4.627	3.065	0.662	0.3	0.0

^(a)^ $\Delta \epsilon_{p}=\left(E_{p}-E_{s}\right)/\left(l+m+n\right)$.

**Figure A1:**
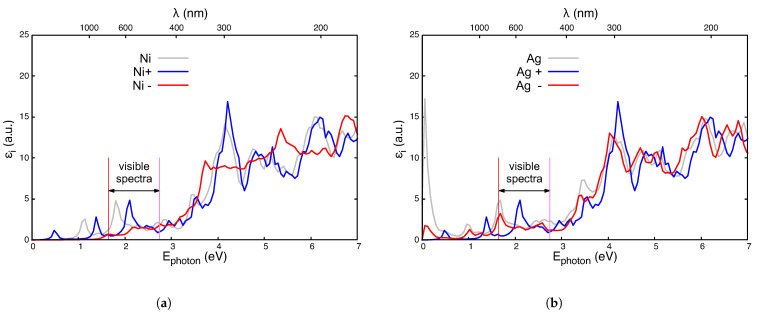
Imaginary part of the dielectric functions $\varepsilon_{i}\left(\omega \right)$ for charged Ti_23_AO_48_ (A = Ni, Ag). (**a**) Neutral Ni (gray line), Ni^+^ (blue line), and Ni^−^ (red line). (**b**) Neutral Ag (gray line), Ag^+^ (blue line), and Ag^−^ (red line). Red and violet vertical lines are included to highlight the visible spectra.

## Data Availability

Data and code availability: Input files for QE can be found at https://drive.google.com/drive/folders/1DOK2i1Vd-v7O8k__ZAg4GfQB1jhXK7x3?usp=share_link accessed on 5 July 2024.

## Abbreviations

The following abbreviations are used in this manuscript:

DFT	Density Functional Theory
DOS	Density of States
GGA	General Gradient Approximation
LDA	Local Density Approximation
NC-PP	Norm-conserving pseudo-potentials
VB	Valence band

## Appendix A. Effect of Charged Impurities

Figure A1a,b show the absorption spectrum for charged Ni and Ag, respectively. The absorption peaks shifts to higher energies for positively charged impurities (Ni^+^, and Ag^+^), but they remain within the visible spectrum. For negatively charged impurities, the Ag absorption peak diminishes but stays at the same energy. However, for Ni^−^ the absorption peak in the visible spectrum disappears.

## References

[1] Rajakumar G., Rahuman A.A., Roopan S.M., Khanna V.G., Elango G., Kamaraj C., Zahir A.A., Velayutham K. (2012). Fungus-mediated biosynthesis and characterization of TiO_2_ nanoparticles and their activity against pathogenic bacteria. Spectrochim. Acta Part A Mol. Biomol. Spectrosc..

[2] Daimon T., Hirakawa T., Kitazawa M., Suetake J., Nosaka Y. (2008). Formation of singlet molecular oxygen associated with the formation of superoxide radicals in aqueous suspensions of TiO_2_ photocatalysts. Appl. Catal. A Gen..

[3] Miyasaka T. (2011). Toward Printable Sensitized Mesoscopic Solar Cells: Light-Harvesting Management with Thin TiO_2_ Films. J. Phys. Chem. Lett..

[4] Dutta P.K., Ginwalla A., Hogg B., Patton B.R., Chwieroth B., Liang Z., Gouma P., Mills M., Akbar S. (1999). Interaction of Carbon Monoxide with Anatase Surfaces at High Temperatures: Optimization of a Carbon Monoxide Sensor. J. Phys. Chem. B.

[5] Hou X., Huang M., Wu X., Liu A. (2009). First-principles calculations on implanted TiO_2_ by 3d transition metal ions. Sci. China Ser. G Phys. Mech. Astron..

[6] Abd A., Habubi N., Alkaim A. (2020). Fe doped TiO_2_ thin films for Solar Cell Applications. Int. J. Adv. Sci. Technol..

[7] Yamaguchi M., Takamoto T., Khan A., Imaizumi M., Matsuda S., Ekins-Daukes N.J. (2005). Super-high-efficiency multi-junction solar cells. Prog. Photovolt. Res. Appl..

[8] Umebayashi T., Yamaki T., Itoh H., Asai K. (2002). Analysis of electronic structures of 3d transition metal-doped TiO_2_ based on band calculations. J. Phys. Chem. Solids.

[9] Andersen O.K. (1975). Linear methods in band theory. Phys. Rev. B.

[10] Schwarz K., Blaha P. (2003). Solid state calculations using WIEN2k. Comput. Mater. Sci..

[11] Diebold U. (2003). The surface science of titanium dioxide. Surf. Sci. Rep..

[12] German E., Faccio R., Mombrú A.W. (2017). A DFT+U study on structural, electronic, vibrational and thermodynamic properties of TiO_2_ polymorphs and hydrogen titanate: Tuning the Hubbard ’U-term’. J. Phys. Commun..

[13] Wang Y., Zhang R., Li J., Li L., Lin S. (2014). First-principles study on transition metal-doped anatase TiO_2_. Nanoscale Res. Lett..

[14] Zhao J., Wu H.C., Li S.H., Lin S.W. (2012). Effect of Fe Concentration on Fe-Doped Anatase TiO_2_ from GGA+U Calculations. Int. J. Photoenergy.

[15] Park S.G., Magyari-Köpe B., Nishi Y. (2010). Electronic correlation effects in reduced rutile TiO_2_ within the LDA+U method. Phys. Rev. B.

[16] Soler J.M., Artacho E., Gale J.D., García A., Junquera J., Ordejon P., Sánchez-Portal D. (2002). The SIESTA method for ab initio order-N materials simulation. Phys. Condens. Matter.

[17] Giannozzi P., Andreussi O., Brumme T., Bunau O., Nardelli M.B., Calandra M., Car R., Cavazzoni C., Ceresoli D., Cococcioni M. (2017). Advanced capabilities for materials modelling with QUANTUM ESPRESSO. J. Phys.-Condens. Mat..

[18] Perdew J.P., Burke K., Ernzerhof M. (1996). Generalized Gradient Approximation Made Simple. Phys. Rev. Lett..

[19] Troullier N., Martins J.L. (1991). Efficient pseudopotentials for plane-wave calculations. Phys. Rev. B.

[20] Kleinman L., Bilander D.M. (1982). Efficacious Form for Model Pseudopotentials. Phys. Rev. Lett..

[21] Aguilera-Granja F., Longo R.C., Gallego L.J., Vega A. (2010). Structural and magnetic properties of X_2_Y (X, Y = Fe, Co, Ni, Ru, Rh, Pd, and Pt) nanoalloys. J. Chem. Phys..

[22] Alonso-Lanza T., Ayuela A., Aguilera-Granja F. (2016). Substitutional 4d and 5d impurities in graphene. Phys. Chem. Chem. Phys..

[23] del Toro R.A., Aguilera-Granja F., Vogel E. (2019). Structural and electronic properties of (_TiO2_)_10_ clusters with impurities: A density functional theory investigation. J. Phys. Chem. Solids.

[24] Hou D.L., Meng H.J., Jia L.Y., Ye X.J., Zhou H.J., Li X.L. (2007). Impurity concentration study on ferromagnetism in Cu-doped TiO_2_ thin films. Europhys. Lett..

[25] Ahmed S.A. (2017). Structural, optical, and magnetic properties of Cu-doped TiO_2_ samples. Cryst. Res. Technol..

[26] Zhao Z.Y., Liu Q.L., Dai W.W. (2016). Structural, Electronic and Optical Properties of BiOX1 xYx (X, Y = F, Cl, Br and I) Solid Solutions from DFT Calculations. Sci. Rep..

[27] Kumaravel V., Rhatigan S., Mathew S., Michel M.C., Bartlett J., Nolan M., Hinder S.J., Gascó A., Ruiz-Palomar C., Hermosilla D. (2020). Mo doped TiO_2_: Impact on oxygen vacancies, anatase phase stability and photocatalytic activity. J. Phys. Mater..

[28] Kumaravel V., Bianchetti E., Mathew S., Hinder S.J., Bartlett J., Di Valentin C., Pillai S.C. (2021). New Insights into Crystal Defects, Oxygen Vacancies, and Phase Transition of Ir-TiO_2_. J. Phys. Chem. C.

[29] Aguilera-Granja F., Aguilera–del–Toro R., Vogel E., Cisternas E. (2021). TiO_2_ nano-clusters adsorbed on surfaces: A density-functional-theoretic study. J. Phys. Chem. Solids.

[30] Alam M.S., Saiduzzaman M., Biswas A., Ahmed T., Sultana A., Hossain K.M. (2022). Tuning bandgap and enhancing optical functions of AGeF3 (A = K, Rb) under pressure for improved optoelectronic applications. Sci. Rep..

[31] Ehrenreich H., Cohen M.H. (1959). Self-Consistent Field Approach to the Many-Electron Problem. Phys. Rev..

[32] Tsafack T., Piccinini E., Lee B.S., Pop E., Rudan M. (2011). Electronic, optical and thermal properties of the hexagonal and rocksalt-like Ge_2_Sb_2_Te_5_ chalcogenide from first-principle calculations. J. Appl. Phys..

[33] Eddiouane A., Chaib H., Nafidi A., Najjaoui M., Ait-Taleb T. (2018). First principles investigation of electronic properties and high refractive index of rutile TiO_2_ for photovoltaic applications. AIP Conf. Proc..

[34] Thatribud A. (2019). Electronic and optical properties of TiO2 by first-principle calculation (DFT-GW and BSE). Mater. Res. Express.

[35] Freysoldt C., Grabowski B., Hickel T., Neugebauer J., Kresse G., Janotti A., Van de Walle C.G. (2014). First-principles calculations for point defects in solids. Rev. Mod. Phys..

[36] Lyons J.L., Van de Walle C.G. (2017). Computationally predicted energies and properties of defects in GaN. NPJ Comput. Mater..

